# Transcriptome responses to aluminum stress in roots of aspen (*Populus tremula*)

**DOI:** 10.1186/1471-2229-10-185

**Published:** 2010-08-23

**Authors:** Nadine Grisel, Stefan Zoller, Marzanna Künzli-Gontarczyk, Thomas Lampart, Martin Münsterkötter, Ivano Brunner, Lucien Bovet, Jean-Pierre Métraux, Christoph Sperisen

**Affiliations:** 1 Swiss Federal Institute for Forest, Snow and Landscape Research (WSL), Zürcherstrasse 111, CH-8903 Birmensdorf, Switzerland; 2 Functional Genomics Center Zurich, University of Zurich and Swiss Federal Institute of Technology Zurich, Winterthurerstrasse 190, CH-8057 Zurich, Switzerland; 3 Institute of Chemistry and Biological Chemistry, Zurich University of Applied Sciences, School of Life Sciences and Facility Management, Einsiedlerstrasse 31, CH-8820 Wädenswil, Switzerland; 4 Institute of Bioinformatics and System Biology, Helmholtz Center Munich, Ingolstädter Landstraße 1, D-85764 Neuherberg, Germany; 5 Department of Biology, University of Fribourg, Chemin du Musée 10, CH-1700 Fribourg, Switzerland; 6 Genetic Diversity Centre, Swiss Federal Institute of Technology, Universitätstrasse 16, CH-8092 Zurich, Switzerland; 7 Dualsystems Biotech AG, Grabenstrasse 11a, CH-8952 Schlieren, Switzerland; 8 Philip Morris International Research & Development, Philip Morris Products SA, Quai Jeanrenaud 56, CH-2000 Neuchâtel, Switzerland

## Abstract

**Background:**

Ionic aluminum (mainly Al^3+^) is rhizotoxic and can be present in acid soils at concentrations high enough to inhibit root growth. Many forest tree species grow naturally in acid soils and often tolerate high concentrations of Al. Previously, we have shown that aspen (*Populus tremula*) releases citrate and oxalate from roots in response to Al exposure. To obtain further insights into the root responses of aspen to Al, we investigated root gene expression at Al conditions that inhibit root growth.

**Results:**

Treatment of the aspen roots with 500 μM Al induced a strong inhibition of root growth within 6 h of exposure time. The root growth subsequently recovered, reaching growth rates comparable to that of control plants. Changes in gene expression were determined after 6 h, 2 d, and 10 d of Al exposure. Replicated transcriptome analyses using the Affymetrix poplar genome array revealed a total of 175 significantly up-regulated and 69 down-regulated genes, of which 70% could be annotated based on *Arabidopsis *genome resources. Between 6 h and 2 d, the number of responsive genes strongly decreased from 202 to 26, and then the number of changes remained low. The responses after 6 h were characterized by genes involved in cell wall modification, ion transport, and oxidative stress. Two genes with prolonged induction were closely related to the *Arabidopsis *Al tolerance genes *ALS3 *(for Al sensitive 3) and *MATE *(for multidrug and toxin efflux protein, mediating citrate efflux). Patterns of expression in different plant organs and in response to Al indicated that the two aspen genes are homologs of the *Arabidopsis ALS3 *and *MATE*.

**Conclusion:**

Exposure of aspen roots to Al results in a rapid inhibition of root growth and a large change in root gene expression. The subsequent root growth recovery and the concomitant reduction in the number of responsive genes presumably reflect the success of the roots in activating Al tolerance mechanisms. The aspen genes *ALS3 *and *MATE *may be important components of these mechanisms.

## Background

Acid soils are prevalent in many regions of the world and present a range of stresses to plants. One of the major stresses caused by these soils is aluminum (Al), which is solubilized by the acidity into the soil solution. Soluble Al exists in its most toxic form as Al^3+^, which can inhibit root growth in many plant species at micromolar concentrations. The resulting reduced and damaged root system limits the capacity of plants to uptake water and nutrients, and increases their susceptibility to other stresses.

The mechanisms by which Al inhibits root growth are not well understood, despite extensive physiological investigations. The root apex is the most sensitive part of the root to Al because it is the site of cell division and cell elongation [1,2]. Since Al is so reactive, it can interact with multiple structures in the apoplasm and symplasm of root cells. In the cell wall, Al primarily binds to the pectin matrix and thereby alters the physical properties of the cell wall [3,4]. In the symplasm, sites of Al interaction include membrane constituents, ion channels, metabolic enzymes, components of signaling pathways, members of the cytoskeleton, and the DNA [3,5]. Although some of the resulting cellular alterations have been proposed to affect cell division or cell elongation, a recent study conducted in *Arabidopsis thaliana *indicates that it is not Al toxicity that is directly responsible for the inhibition of root growth. Genetic and biochemical evidence suggest that the cells of the root apex have an ATR-controlled mechanism to monitor Al-induced DNA damage [6]. In plants exposed to Al, this mechanism activates blockage of cell cycle progression and thus root growth. This active response of roots to Al may not protect individual plants, but it may help to safeguard plant populations by preventing the passage of damaged DNA to subsequent plant generations [6].

Plant species vary considerably in their degree of Al tolerance, and even genotypes within the same plant species vary in their ability to cope with Al. The mechanisms providing enhanced Al tolerance can be classified into external and internal mechanisms [5,7]. The best-documented external mechanism is the efflux of organic acid anions, such as malate, citrate, and oxalate, from the roots in response to Al exposure [8]. These organic acid anions effectively chelate Al and thereby detoxify Al in the rhizosphere. Other proposed external mechanisms involve secretion of proteins [9], root-mediated increase of the rhizosphere pH [10], and masking Al binding sites at the cell wall [11,12]. Proposed internal tolerance mechanisms include chelation of Al by organic acid anions or phenolic compounds and sequestration of Al in the vacuole [5].

The genes responsible for the Al-induced efflux of malate and citrate have been recently isolated and demonstrated to represent major genes for Al tolerance in several plant species [8]. The genes responsible for the efflux of malate belong to the *ALMT *(for Al-activated malate transporter) gene family [13-15] and those involved in the efflux of citrate to the *MATE *(for multidrug and toxin efflux protein) gene family [16-20]. All these genes encode membrane proteins, consistent with their role in facilitating the efflux of organic acid anions. Additional genes with putative roles in Al tolerance have been identified in Al-sensitive mutants of rice (*Oryza sativa*) and *Arabidopsis*. The rice mutants *star1 *(for sensitive to Al rhizotoxicity 1) and *star2 *were found to be mutated in genes encoding two proteins that form together an ATP-binding cassette (ABC) transporter [21]. This complex mediates the transport of UDP-glucose to the cell wall, where the molecule is believed to play a role in masking Al binding sites. Similarly to *star1 *and *star2*, the Al-sensitive mutants *als1 *(for Al sensitive 1) and *als3 *of *Arabidopsis *are mutated in genes encoding ABC transporter-like proteins [22,23]. Although the substrate of these proteins is not known, the mutant phenotypes and patterns of gene expression have led to the proposal that the two proteins transport and sequester Al to confer Al tolerance. ALS1 is believed to be involved in the intracellular transport of Al to the vacuole [22], whereas ALS3 appears to be necessary for the long-distance transport of Al to the aerial parts of the plant [23].

Further insight into the molecular mechanisms involved in Al toxicity and tolerance come from gene expression analyses. Genome-wide transcriptome analyses in roots of *Arabidopsis *have revealed a number of cellular processes that are altered in response to Al exposure. Examples are cell wall modification, protein metabolism, transport processes, and oxidative stress [24,25]. In maize (*Zea mays*), wheat (*Triticum aestivum*), and *Medicago truncatula*, gene expression was analyzed in plant lines with contrasting levels of Al tolerance [26-28]. These studies have led to the identification of several candidate genes for Al tolerance, including previously identified genes encoding organic acid efflux transporters, genes controlling levels of reactive oxygen species (ROS), as well as genes involved in pectin modification and immobilization of Al by phosphate.

Forest trees generally tolerate high concentrations of Al [29]. For example, seedlings of Norway spruce (*Picea abies*) and birch *(Betula pendula*) did not show any reduction in root growth at Al concentrations below 0.3 and 3 mM, respectively [30,31]. In contrast, Al concentrations as low as 50 μM tend to affect the root growth of *Arabidopsis *and several crop plants (e.g. [24,28,32]). Since many forest tree species grow naturally in acid soils, it appears likely that such species have developed adaptive mechanisms that enable them to tolerate high Al conditions. Analyses of the root responses to Al in forest trees may thus broaden our understanding of Al tolerance mechanisms in plants.

In a previous study, we used clonal aspen (*Populus tremula*, clone Birmensdorf) to investigate Al-induced efflux of organic acid anions from roots [33]. The results showed that Al concentrations ≥ 200 μM induced the efflux of citrate, while Al concentrations ≥ 500 μM enhanced the efflux of oxalate. At these concentrations, Al did not cause any visible symptoms at the root tips, indicating that the aspen clone examined tolerates high concentrations of Al. Using the same aspen clone, we investigated temporal patterns of root gene expression under Al stress. Changes in gene expression were assessed by application of the Affymetrix poplar genome array. The expression of selected genes was further analyzed by reverse-transcription PCR.

## Results

### Effect of Al on root growth and callose formation

**Figure 1:**
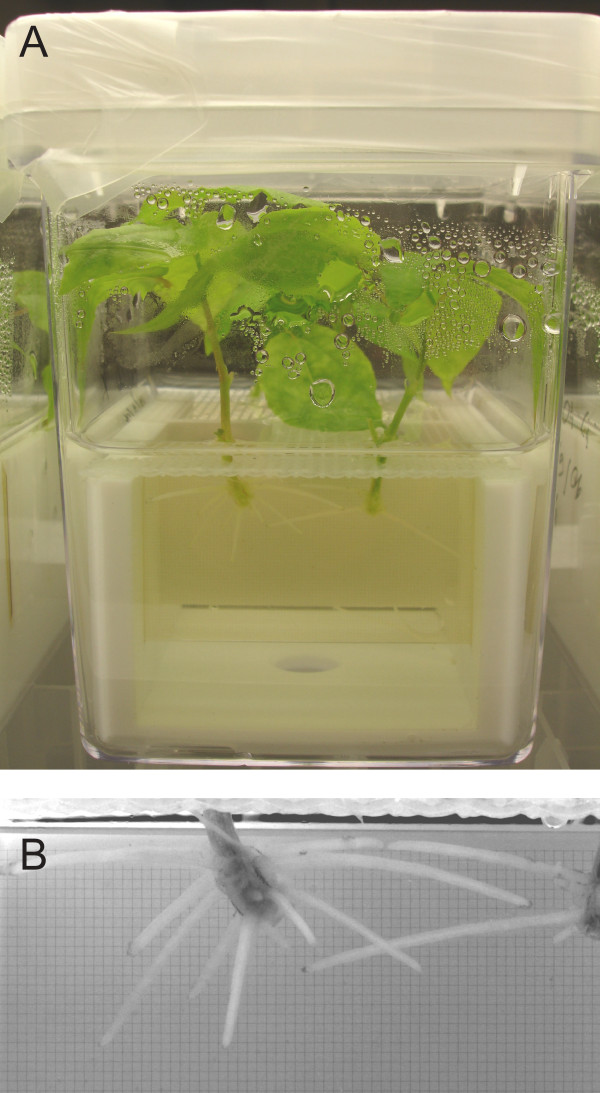
**Solution culture of clonal aspen (*Populus tremula*)**. **A **Solution cultures were established in modified Magenta boxes. **B **Root growth was photographically monitored. Glass slides with a 5 μm grid were used as a reference to measure root growth.

**Figure 2:**
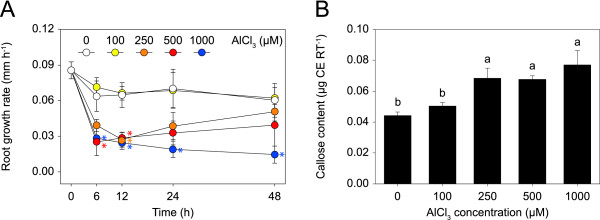
**Effect of Al on root growth and callose formation in aspen**. **A **Time series of the root growth rates of plants treated with no or increasing concentrations of Al for 2 d. Asterisks indicate significant differences between treated and control plants (repeated measures ANOVA; *p *< 0.05). **B **Callose content in root tips (RT) after 2 d of Al treatment. Callose content is expressed as a curdlan equivalent (CE). The means and standard error values from four independent roots are shown. Different letters indicate significant differences between treatments (ANOVA; *p *< 0.05).

To determine plant treatment conditions suitable for gene expression profiling, we examined the effect of Al on root growth. Clonal aspen was treated with either no Al or increasing concentrations of Al up to 1000 μM for 2 d in solution culture (Fig. 1). Exposure of the roots to 100 μM Al did not affect their growth (Fig. 2A). In contrast, 250, 500, and 1000 μM Al caused a rapid and strong inhibition of root growth, with a reduction in growth rate of ≥ 40% observed after 6 h. During prolonged Al exposure, the root growth of plants treated with 250 and 500 μM Al partially recovered, while that of plants treated with 1000 μM further decreased, although not significantly (*p *> 0.1). As a further indicator of Al toxicity, we quantified callose, which accumulates in many plant species upon Al exposure [3]. The content of callose in the root tips increased with Al in the medium, with a significant increase observed at Al concentrations ≥ 250 μM (Fig. 2B).

### Root growth of plants used for gene expression profiling

**Figure 3:**
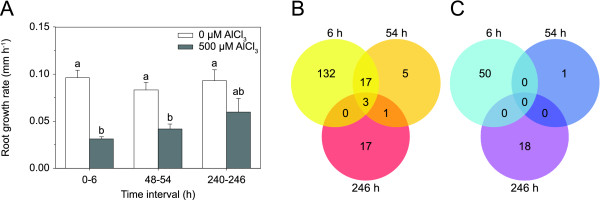
**Aspen genes with significantly altered expression in Al-treated root tips compared to control root tips**. **A **Root growth rates of plants used for the gene expression profiling. Roots were treated with 500 μM Al for 6 h, 54 h, and 246 h. During the 246 h treatment, the culture medium was exchanged every 2 d to maintain a constant Al stress. Means and standard error values from four independent roots are shown. Different letters indicate significant differences between treatments (ANOVA; *p *< 0.05). Number of up-regulated genes (**B**) and down-regulated genes (**C**) after 6 h, 54 h, and 246 h of Al treatment (≥ 2-fold change in expression).

Based on the results of the dose-response experiment, plants were treated with 500 μM Al for gene expression profiling. Treatment with 500 μM was preferred over 1000 μM, which tended to cause necrotic lesions at the root tips. The time points selected were 6 h, 54 h (designated as 2 d), and 246 h (10 d). The 6 h time point marked the rapid Al-induced inhibition of root growth. The 2 and 10 d time points represented the period of root growth recovery. Since some plant genes are regulated by a diurnal rhythm and circadian clock, the exact duration of the treatments were designed such that the roots could be sampled at the same time each. During the 10 d treatment, the culture medium was exchanged every 2 d to maintain a constant Al stress. Consistent with the results of the dose-response experiment, Al induced a rapid inhibition of root growth (68% reduction at 6 h; Fig. 3A). The growth of roots treated with Al for 2 and 10 d gradually recovered (50% reduction at 2 d; 36% reduction at 10 d). Al concentrations of the culture medium decreased only slightly during the 2 d treatment periods (on average by 36.7 ± 13.9 μM), and the pH remained constant (pH 4.0 ± 0.1). Therefore, the increase in root growth could not be explained by either a possible decrease of Al in the culture medium due to the uptake of Al by the plants, or altered Al speciation due to pH change.

### Changes in gene expression

Gene expression profiles were determined using the Affymetrix poplar genome array, interrogating over 56,000 transcripts and gene predictions. To detect genes that are significantly regulated by Al, we employed an approach that allowed an estimation of the false discovery rate (FDR) in multiple testing. The *q*-value, which is a positive FDR analogue of the *p*-value [34], was set to 0.15. As a further criterion, we used a two-fold change cut-off. By these criteria and after removing redundant probe sets, a total of 244 genes were differentially expressed. Treatment of the roots for 6 h resulted in the up-regulation of 152 genes and the down-regulation of 50 genes (Fig. 3B; C). These numbers decreased significantly when the roots were treated for 2 d (26 genes up-regulated, 1 down-regulated). Of the induced genes at this time point, 20 were also induced after 6 h. Treatment of the roots for 10 d yielded a similar low number of responsive genes (21 genes up-regulated, 18 down-regulated). Three genes were induced across all three time points.

### Validation of microarray data

**Figure 4:**
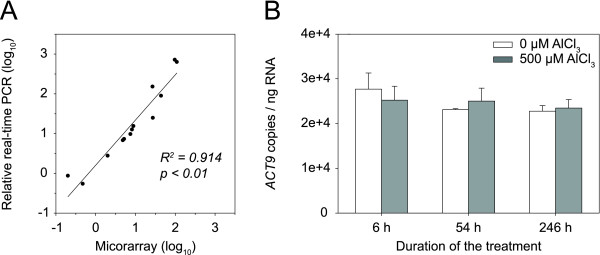
**Validation of microarray data**. **A **Relationship between microarray and qRT-PCR data. Transcript levels of eight differentially expressed genes were quantified by qRT-PCR relative to *ACT9*. The fold changes in gene expression in response to Al treatment were log_10 _transformed. The microarray data were plotted against the qRT-PCR data. **B **Expression levels of *ACT9 *in the root samples used for microarray experiments. *ACT9 *transcript levels did not significantly change during the 10 d Al treatment (ANOVA; *p *> 0.6). The means and standard error values from three independent samples are shown.

The microarray data were independently verified by real-time reverse transcription PCR (qRT-PCR). Eight genes were analyzed, displaying a wide range of expression profiles. Transcripts were quantified relative to the actin 9 (*ACT9*) gene, which was isolated and sequenced in this study. The qRT-PCR analyses were performed with RNA from the microarray experiment (18 samples) and with RNA from an independent Al treatment experiment (2 samples). Six genes were tested with the RNA from both experiments. A significant correlation was found between the microarray and qRT-PCR data (*R*^2 ^= 0.91; *p *< 0.01; Fig. 4A). The magnitude of the relative changes in transcript abundance did not differ greatly between the two techniques. Exceptions were two strongly induced genes encoding a basic pathogenesis-related protein and a family 3 sulfate transporter: expression differences measured by qRT-PCR were 10 times greater than those measured by microarrays, probably due to the fact that qRT-PCR has a wider dynamic range than microarrays [35]. To examine the validity of using *ACT9 *as a reference gene, absolute qRT-PCR was performed. The analysis showed that the expression of the gene did not change significantly during the 10 d treatment (*p *> 0.6; Fig. 4B).

### Annotation and functional categorization of Al-responsive genes

BLASTX searches against the *Arabidopsis *protein database allowed 172 of the responsive genes to be annotated (expectation (*E*)-value ≤ 1 × 10^-10^). Additional 29 genes had matches to *Arabidopsis *genes with unknown functions, and 44 genes had no detectable similarity to *Arabidopsis *proteins (*E*-value ≥ 1 × 10^-4^). A complete list of the responsive genes is presented in additional file 1.

**Figure 5:**
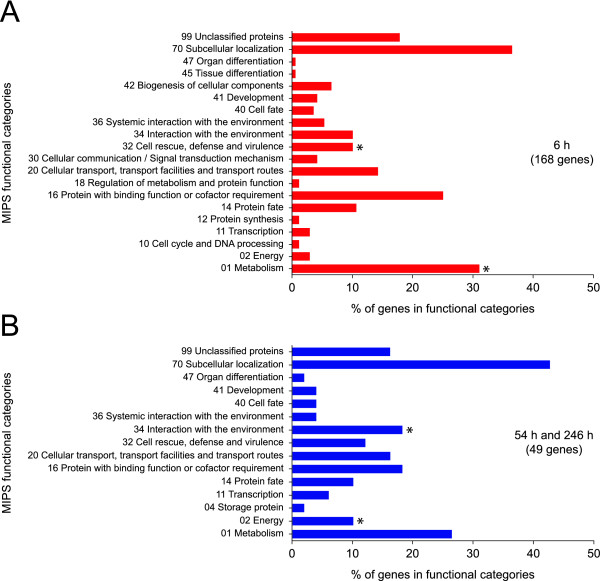
**Functional categorization of the differentially expressed aspen genes using the Munich Information Center for Protein Sequences (MIPS) functional catalogue**. Categories of the differentially expressed genes after 6 h of Al exposure (**A**), and the combined set of genes differentially expressed after 2 and 10 d of Al exposure (**B**). Categories whose members were enriched are indicated by asterisks (*p *< 0.05, FDR < 0.05).

To identify biological processes, the genes with known functions were classified using the Munich Information Center for Protein Sequences (MIPS) functional catalogue [36]. The genes present on the microarray were also classified to allow identification of categories whose genes were over-represented compared to the genes present on the microarray. These analyses were carried out for the genes regulated after 6 h and for the combined set of genes regulated after 2 and 10 d. Top-level categories and subcategories with an enrichment *p*-value < 0.05 and a FDR < 0.05 were regarded as pertinent to the time points examined. By these criteria, genes assigned to the top-level categories 'metabolism' and 'cell rescue and defense' were enriched after 6 h (Fig. 5A; additional file 2). Within the 'metabolism' category, the genes related to 'carbohydrate metabolism' were enriched and included several genes involved in cell wall modification (additional file 2). Additional enriched genes related to cell wall modification were assigned to the top-level category 'biogenesis of cellular components'. The top-level category 'cell rescue and defense' included a number of enriched genes related to 'oxidative stress response' and 'detoxification'. An additional major group of enriched genes had functions in 'ion transport'. Smaller groups of enriched genes were related to 'cell death' and 'plant signaling molecules'. Genes implicated in 'ion transport' and 'carbohydrate metabolism' were also enriched during prolonged Al exposure, but their number was small (additional file 2). Additional genes enriched during prolonged Al exposure were assigned to the top-level categories 'energy' and 'interaction with the environment' (Fig. 5B).

### Genes related to cell wall modification

All the genes recorded that are related to cell wall modification were regulated after 6 h of Al exposure (Table 1). Nine of these genes have functions in the modification of pectin. A pectin methylesterase gene, two pectate lyase genes, and two galacturonosyltransferase genes were down-regulated. Of the three pectin methylesterase inhibitor genes identified, two were down-regulated and the other induced. Additional genes encoded proteins that target cellulose and xyloglucan. The three endo-1,4-β-glucanase and three xyloglucan endotransglucosylase/hydrolase genes recorded showed contrasting patterns of up- and down-regulated expression.

**Table 1:** Selected genes differentially expressed in Al-treated aspen roots.

				Fold change		
**Affymetrix probe set ID**^**a**^	**Gene model name**^**b**^	**AGI n°**^**c**^	**Annotation of best hit in *Arabidopsis thaliana *genome**^**d**^	6 h	2 d	10 d
**Cell wall^e^**						
PtpAffx.21218.1.S1_at	eugene3.00280166	AT3G13750	Beta-galactosidase 1 (BGAL1; GH35)	0.2	0.5^ns^	0.5^ns^
PtpAffx.122394.1.S1_at	gw1.I.26.1	AT5G64570	Beta-xylosidase 4 (XYL4; GH3)	0.2	0.8^ns^	1.3^ns^
PtpAffx.212738.1.S1_at	estExt_fgenesh4_pg.C_LG_XV0425	AT5G62620	Galactosyltransferase family protein	2.1	1.0^ns^	1.2^ns^
Ptp.7955.1.S1_at	gw1.VIII.37.1	AT1G24170	Galacturonosyltransferase-like 8 (GATL8; GT9)	0.5	0.7^ns^	1.0^ns^
PtpAffx.208384.1.S1_at	gw1.VIII.37.1	AT1G70090	Galacturonosyltransferase-like 9 (GATL9; GT8)	0.5	0.8^ns^	1.1^ns^
PtpAffx.209224.1.S1_at	fgenesh4_pg.C_LG_X001601	AT1G65610	Endo-1,4-beta-glucanase Korrigan 2 (KOR2; GH9)	2.1	1.3^ns^	1.0^ns^
PtpAffx.207811.1.S1_at	estExt_fgenesh4_pg.C_LG_VIII0680	AT1G65610	Endo-1,4-beta-glucanase Korrigan 2 (KOR2; GH9)	2.9	1.3^ns^	1.0^ns^
Ptp.4073.1.S1_s_at	estExt_fgenesh4_pg.C_LG_XIV0665	AT4G02290	Endo-1,4-beta-glucanase 17 (GH9)	0.2	0.5^ns^	1.0^ns^
PtpAffx.116752.1.A1_at	gw1.VIII.287.1	AT3G26380	Glycosyl hydrolase family protein 27 (GH27)	2.0	1.6	1.4^ns^
PtpAffx.20309.1.S1_at	estExt_fgenesh4_pg.C_LG_X2099	AT5G04500	Glycosyl transferase family protein 64 (GT64)	5.2	3.8	1.7^ns^
Ptp.4642.1.S1_at	estExt_fgenesh4_pg.C_LG_XI1340	AT1G30620	UDP-D-xylose 4-epimerase 1 (UXE1)	2.2	1.7	1.1^ns^
PtpAffx.31211.1.A1_at	eugene3.00140929	AT1G05560	UDP-glucose transferase 1 (UGT1; GT1)	6.4	0.8^ns^	1.5^ns^
Ptp.160.1.S1_x_at	fgenesh4_pm.C_LG_II000873	AT3G62830	UDP-glucuronic acid decarboxylase 2 (UXS2)	2.2	1.2^ns^	1.3^ns^
PtpAffx.119179.1.A1_at	gw1.1681.2.1	AT5G48070	Xyloglucan endotransglucosylase/hydrolase 20 (XTH20; GH16)	4.3	1.6^ns^	1.7^ns^
Ptp.2467.1.A1_x_at	gw1.XIX.2748.1	AT4G03210	Xyloglucan endotransglucosylase/hydrolase 9 (XTH9; GH16)	0.3	0.6^ns^	1.1^ns^
Ptp.3050.1.S1_s_at	estExt_Genewise1_v1.C_LG_XIV2162	AT1G10550	Xyloglucan:xyloglucosyltransferase 33 (XTH33; GH16)	0.3	0.8^ns^	0.9^ns^
Ptp.4810.1.A1_s_at	estExt_Genewise1_v1.C_LG_III0932	AT1G04680	Pectate lyase family protein	0.5	0.8^ns^	0.9^ns^
PtpAffx.1316.2.S1_s_at	eugene3.00010425	AT1G04680	Pectate lyase family protein	0.2	1.0^ns^	0.9^ns^
PtpAffx.9932.3.S1_a_at	eugene3.00030462	AT1G53830	Pectin methylesterase 2 (PME2)	0.4	0.9^ns^	0.8^ns^
PtpAffx.9932.2.A1_s_at	estExt_fgenesh4_pm.C_290002	AT3G14310	Pectin methylesterase inhibitor 3 (PMEI3)	0.5	1.0^ns^	1.0^ns^
Ptp.7635.1.S1_at	eugene3.00140717	AT1G02810	Pectin methylesterase inhibitor 7 (PMEI7)	4.0	1.5^ns^	0.9^ns^
PtpAffx.207505.1.S1_at	gw1.VIII.1476.1	AT3G10720	Pectin methylesterase inhibitor 25 (PMEI25)	0.3	0.5^ns^	1.0^ns^
Ptp.3290.1.S1_at	gw1.VII.2504.1	AT5G08200	Peptidoglycan-binding LysM domain-containing protein	2.2	1.2^ns^	1.1^ns^
Ptp.2725.1.S1_at	gw1.X.2924.1	AT5G62150	Peptidoglycan-binding LysM domain-containing protein	3.1	1.2^ns^	1.2^ns^
PtpAffx.208179.1.S1_at	eugene3.00081504	AT2G23770	Peptidoglycan-binding LysM domain-containing protein	2.3	1.5^ns^	1.1^ns^
**Transport**						
**Ion transport**						
Ptp.6087.1.S1_at	eugene3.97260001	AT5G01600	Ferritin 1 (FER1)	0.3^ns^	0.4^ns^	0.3
PtpAffx.595.4.S1_s_at	estExt_fgenesh4_pg.C_1470038	AT2G24520	H^+^-ATPase 5 (HA5)	2.3	1.5^ns^	1.6^ns^
PtpAffx.208738.1.S1_s_at	estExt_fgenesh4_pm.C_LG_X0276	AT5G64560	Mg transporter CorA-like family protein (MRS2-2)	4.8	2.2^ns^	1.4^ns^
PtpAffx.46328.1.A1_at	gw1.I.4154.1	AT3G19640	Mg transporter CorA-like family protein (MRS2-3)	3.8	2.8	1.3^ns^
PtpAffx.204370.1.S1_at	fgenesh4_pg.C_LG_IX000025	AT5G44370	Phosphate transporter 4;6 (PHT4;6)	4.3	1.8^ns^	1.1^ns^
PtpAffx.249.377.A1_at	fgenesh4_pm.C_LG_V000517	AT3G51895	Sulfate transmembrane transporter 3;1 (SULTR3;1)	2.4	1.3^ns^	1.0^ns^
PtpAffx.63924.1.S1_at	eugene3.01570002	AT5G19600	Sulfate transmembrane transporter 3;5 (SULTR3;5)	26.8	109.2	3.2^ns^
PtpAffx.46298.1.S1_at	estExt_fgenesh4_pg.C_LG_VIII0032	AT5G55630	Two pore K^+ ^channel 1 (TPK1)	2.1	1.3^ns^	1.1^ns^
**Transport facilities**						
PtpAffx.119032.1.S1_s_at	gw1.XVI.2587.1	AT2G37330	Aluminum sensitive 3 (ALS3)	44.4	27.5	5.2
Ptp.5171.1.S1_at	gw1.VI.655.1	AT5G03910	ABC transporter homolog 12 (ATH12)	2.5	1.4^ns^	1.1^ns^
PtpAffx.204839.1.S1_at	gw1.IX.3299.1	AT3G08040	MATE (FRD3)	8.9	4.2^ns^	1.7^ns^
Ptp.2869.1.A1_at	gw1.I.5916.1	AT1G30690	SEC14 cytosolic factor family protein/phosphoglyceride transfer family protein	0.3	0.8^ns^	0.9^ns^
**Oxidative stress response^f^**						
Ptp.2903.1.A1_s_at	gw1.XII.485.1	AT3G22370	Alternative oxidase 1A (AOX1A)	8.6	1.5^ns^	3.6^ns^
PtpAffx.56141.1.A1_at	grail3.0007029701	AT5G20230	Blue copper binding protein (BCB)	2.4	1.4^ns^	2.4^ns^
PtpAffx.153878.1.A1_at	gw1.XV.2559.1	AT5G51100	Fe superoxide dismutase (FSD2)	1.4^ns^	1.5^ns^	2.8
PtpAffx.134361.1.A1_s_at	eugene3.00031141	AT1G64500	Glutaredoxin family protein	0.4	0.7	0.4^ns^
PtpAffx.2286.4.S1_a_at	-	AT1G17180	Glutathione S-transferase tau 25 (GSTU25)	0.4	0.5^ns^	1.9^ns^
PtpAffx.23427.1.S1_s_at	estExt_fgenesh4_pg.C_LG_VIII1530	AT2G29420	Glutathione S-transferase tau 7 (GSTU7)	2.9	1.0^ns^	1.5^ns^
PtpAffx.29337.1.A1_at	eugene3.00030584	AT5G67400	Peroxidase 73 (P73)	0.5	1.0^ns^	1.0^ns^
PtpAffx.36879.1.A1_s_at	gw1.VII.698.1	AT5G24070	Peroxidase family protein	0.5	0.7^ns^	0.8^ns^
PtpAffx.43372.1.A1_at	fgenesh4_pg.C_LG_XVI000455	AT5G06720	Peroxidase, putative	3.1	1.4^ns^	0.9^ns^
PtpAffx.54628.1.S1_at	estExt_fgenesh4_pg.C_LG_XVI1240	AT5G05340	Peroxidase, putative	13.3	1.7^ns^	1.1^ns^
PtpAffx.55376.1.S1_at	fgenesh4_pg.C_LG_XIV000840	AT5G05340	Peroxidase, putative	2.0^ns^	0.4^ns^	0.1

^a^Affymetrix probe set identifier (ID) of the GeneChip Poplar Genome Array 61 K (Affymetrix).

^b^Preferred gene model name attributed by Poparray v1.2 http://aspendb.uga.edu/poparray.

^c^*Arabidopsis *genome identifier (AGI n°).

^d^Annotation of best hit in *Arabidopsis *genome with a *E*-value cut-off of < 0.05.

^e^C-compound and carbohydrate metabolism and cell wall.

^f^Oxidative stress response and detoxification.

^ns^not significantly up- or down-regulated.

### Genes related to ion transport

The regulated genes encoding ion transporters included both cation and anion transporters. With the exception of a ferritin gene, all the ion transporters were induced. A two-pore K^+ ^channel gene was closely related to the *Arabidopsis *gene *TPK1*, which is a key regulator of K homeostasis [37]. Two genes encoded CorA-like Mg transporters, belonging to a class of transmembrane proteins that are suggested to function in the uptake and intracellular transport of Mg [38]. The anion transporters were composed of a family 4 phosphate transporter and two family 3 sulfate transporter genes. The encoded family 4 phosphate transporter was related to the *Arabidopsis *protein PHT4;6, proposed to be involved in the biosynthesis of cell wall polysaccharides [39]. One of the encoded sulfate transporters was related to the *Arabidopsis *SULTR3;5, a protein functioning in the root-to-shoot transport of sulfate [40].

### Genes related to oxidative stress

Three of the regulated genes related to oxidative stress belong to the network of genes that control levels of ROS [41]. An alternative oxidase and a Fe superoxide dismutase gene were up-regulated, while a glutaredoxin gene was down-regulated. Additional genes encoded tau-type glutathione *S*-transferases and peroxidases. Individual members of these gene families showed contrasting patterns of up- and down-regulated expression.

### Identification of putative Al tolerance genes

Among the regulated genes encoding transport facilitators, there were two genes that may play a role in Al tolerance. One was related to the *Arabidopsis *Al tolerance gene *ALS3 *[23]. The other belonged to the *MATE *gene family and was related to the *Arabidopsis *citrate efflux transporter gene *MATE *[18] and to *FRD3 *(for ferric reductase defective 3), which encodes a citrate transporter responsible for loading Fe into the xylem [42]. Both aspen genes were induced: *ALS3 *was up-regulated at all three time points, and *MATE *at 6 h and 2 d.

To examine whether the genes identified by microarrays are indeed genes related to the *Arabidopsis ALS3 *and *MATE*, RT-PCR was performed and the PCR products were sequenced. In the case of *ALS3*, the entire coding sequence was isolated. The predicted protein shared 79% sequence identity with the *Arabidopsis *ALS3 [23] and 71% with STAR2 of rice [21]. All of these genes encode the transmembrane domain of ABC transporters and lack the ATP-binding cassette domain. In the case of *MATE*, approximately 80% was isolated. The predicted protein shared 60% sequence identity with the *Arabidopsis *MATE [18] and 62% with FRD3 [43]. Similar degrees of sequence identities were found between the aspen MATE and MATE of sorghum (54%) [16], barley (54%) [17], and maize (56%) [20].

### Expression of *ALS3 *and *MATE*

**Figure 6:**
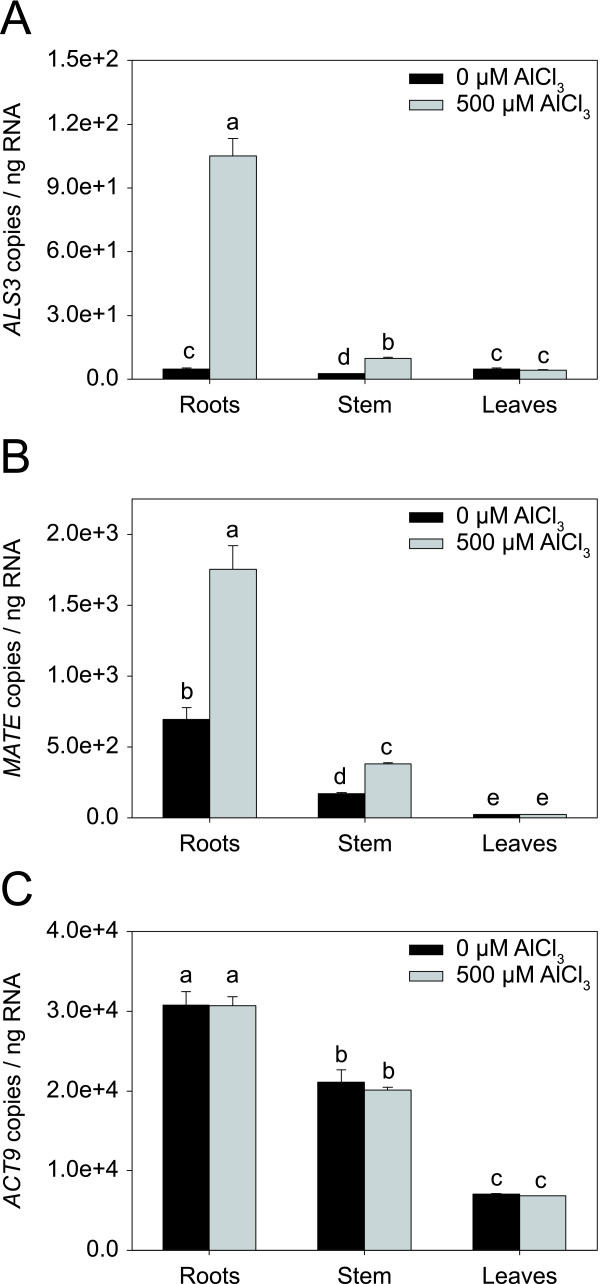
**Expression levels of *ALS3, MATE*, and *ACT9 *in different aspen tissues**. Expression levels of *ALS3 *(**A**), *MATE *(**B**), and *ACT9 *(**C**) in root, stem, and leaf tissue of plants treated with no or 500 μM Al for 2 d. Transcript levels were quantified by absolute qRT-PCR. The means and standard error values from three independent samples are shown. Different letters indicate significant differences between treatments and tissues (ANOVA; *p *< 0.05).

Each of the three *Arabidopsis *genes *ALS3*, *MATE*, and *FRD3 *are characterized by a distinct pattern of expression. Under non-stressed conditions, *ALS3 *is expressed in the phloem throughout the plant and in the root epidermis [23]. *MATE *and *FRD3 *are primarily expressed in the root [18,43]. Exposure to Al strongly induces *ALS3 *and *MATE *in the root, but not *FRD3 *[18,23]. To examine whether the expression of the aspen *ALS3 *and *MATE *is coherent with that of the *Arabidopsis ALS3 *and *MATE*, absolute qRT-PCR was carried out with RNA isolated from root, stem, and leaf tissue of a separate set of plants treated with either no Al or 500 μM Al for 2 d. The results of the analysis showed that *ALS3 *is expressed in all three tissues under non-stress conditions with little differences among the tissues (Fig. 6A). In contrast, *MATE *was more strongly expressed in the root than in the stem and leaves (Fig. 6B). Treatment of the plants with Al led to an induction of both genes (Fig. 6A; B). *ALS3 *was strongly induced in the root (22.3-fold) and to a lower extent in the stem (3.9-fold), while *MATE *was induced both in the root (2.5-fold) and in the stem (2.3-fold). The expression levels of *ACT9 *did not differ between the treatments, but did between the tissues, where they were highest in the root (Fig. 6C). However, the differences in *ACT9 *expression in the different tissues did not explain the tissue-specific expression of *ALS3 *and *MATE*, as was shown by plotting transcript levels relative to *ACT9 *(data not shown). Taken together, expression patterns of the two aspen genes were highly coherent with that of the *Arabidopsis ALS3 *and *MATE*.

**Figure 7:**
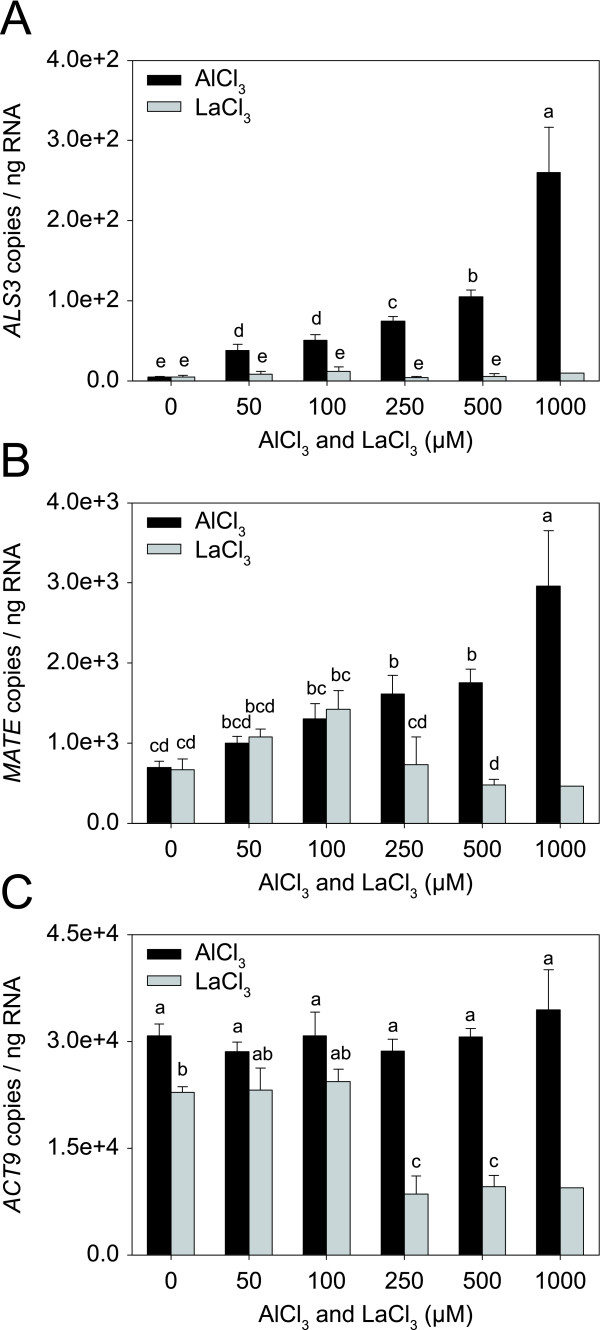
**Expression levels of *ALS3, MATE*, and *ACT9 *in aspen roots treated with Al and La**. Expression levels of *ALS3 *(**A**), *MATE *(**B**) and *ACT9 *(**C**) in the root tips of plants treated with no or increasing Al and La concentrations for 2 d. Transcript levels were quantified by absolute qRT-PCR. The means and standard error values from three independent samples are shown, except for the roots treated with 1000 μM La. Different letters indicate significant differences between treatments (ANOVA; *p *< 0.05).

To examine whether Al induces the two genes in a concentration-dependent way, a separate set of plants were treated with increasing concentrations of Al up to 1000 μM Al. Plants were also treated with increasing concentrations of lanthanum (La), which has chemical properties similar to those of Al and is known to inhibit root growth [44]. Root growth measurements showed that La inhibited root growth in a similar way to Al, but the inhibition was stronger (data not shown). Absolute qRT-PCR showed that the expression of both genes increased with Al in the medium (Fig. 7A; B). In contrast, exposure of the roots to La induced *MATE*, but not *ALS3 *(Fig. 7A; B). Based on levels of *ACT9 *expression, it appeared that La concentrations ≥ 250 μM, affected transcription (Fig. 7C).

## Discussion

In this study, we analyzed temporal patterns of root growth and root gene expression in aspen under Al stress. Two phases of root growth could be distinguished: a rapid Al-induced growth inhibition (within 6 h) and a subsequent growth recovery. From our analysis of gene expression at three time points, we found a pronounced decrease in the number of responsive genes from 202 to 27 between 6 h and 2 d of Al exposure. The number of changes then remained low. Similar patterns of root growth and gene expression were observed in an Al-tolerant line of *Medicago truncatula *[28]. The root growth of this line largely recovered within 2 d of Al exposure, while that of an Al-sensitive line remained inhibited. The number of responsive genes was found to decrease in both lines between 12 h and 2 d of Al exposure, but the reduction was stronger in the Al-tolerant line (3.3-fold) than in the Al-sensitive line (1.5-fold). These patterns may reflect the success of roots in activating Al tolerance mechanisms and the restoration of the transcriptome to a prestress program, and in the case of the Al-sensitive line of *Medicago *the failure to establish an adaptive response.

Consistent with the strong Al-induced root growth inhibition at the 6 h time point, a number of differentially expressed genes can be associated with toxic effects of Al. Our enrichment analysis identified cell wall modification, ion transport, and oxidative stress as major biological processes operating at this time point. The regulation of cell wall-related genes was not surprising because the cell wall is considered to be a major site of Al toxicity [4]. Physiological studies show consistently that a large portion of the Al absorbed by roots is localized to the apoplasm [5]. Several studies indicate that the Al bound to the apoplasm can make the cell wall more rigid, thus reducing its extensibility, which is required for normal cell extension [45,46]. Plants are believed to employ several different mechanisms to loosen the cell wall during cell extension. Proposed cell wall loosening agents include expansin, xyloglucan endotransglucosylase/hydrolase (XHT), endo-1,4-β-glucanase, and hydroxyl radicals [47]. In this study, we recorded several genes encoding XHTs and endo-1,4-β-glucanases, with some of the genes being up-regulated and others being down-regulated. These contrasting responses may reflect the different enzymatic functions that members of the XHT and endo-1,4-β-glucanase protein families can fulfill [47]. Although the exact function of the XHT and endo-1,4-β-glucanase genes identified is not known, it appears likely that these genes play a role in minimizing the toxic effects of Al on the cell wall. A number of physiological studies demonstrate that Al strongly interacts with membrane channel proteins, resulting in the disruption of the uptake and homeostasis of cations, such as Ca, Mg, and K [3]. In our study, we recorded up-regulation of a two-pore K^+ ^channel gene and two CorA-like Mg transporter genes, indicating that Al interferes with K and Mg homeostasis in aspen roots. Mg transporters have so far not been found to be Al inducible. Nevertheless, two lines of evidence indicate that Mg transporters play an important role in Al toxicity. The activity of a CorA-like Mg transporter of *Arabidopsis *was shown to be blocked by micromolar concentrations of Al [38]. In addition, the same CorA-like Mg transporter alleviated Al toxicity when overexpressed *in planta *[48]. Although oxidative stress is commonly regarded to be a major component of Al toxicity, we recorded only a small number of genes belonging to the ROS network of genes. A Fe superoxide dismutase and a mitochondrial alternative oxidase gene were up-regulated. While Fe superoxide dismutases are responsible for ROS scavenging [49], alternative oxidases serve to lower mitochondrial ROS formation [50]. Induction of the alternative oxidase is entirely in agreement with the finding that Al can induce ROS formation in mitochondria [51].

Based on information from *Arabidopsis *and crop plants, three differentially expressed aspen genes may play a role in Al tolerance mechanisms. Two genes were closely related to the *Arabidopsis *Al tolerance genes *ALS3 *and *MATE*. Patterns of expression in different plant organs and in response to Al strongly suggest that the two aspen genes are homologs of the *Arabidopsis ALS3 *and *MATE*. The exact function of the *Arabidopsis ALS3 *is not known. Mutant *als3 *seedlings grown in Al-containing medium exhibit a severe arrest of root growth and inhibited leaf expansion [52]. Based on this phenotype and patterns of tissue-specific expression, ALS3 has been proposed to mediate transport of Al away from sensitive root tissues to aerial parts of the plant for sequestration or exudation [23]. The *Arabidopsis ALS3 *is expressed in the phloem throughout the plant and is strongly induced by Al in the root cortex [23]. Our aspen *ALS3 *gene was expressed in the root, stem, and leaves, and was strongly induced by Al in the root. In addition, the aspen *ALS3 *was inducible by Al, but not by La. This is consistent with the finding that the *Arabidopsis *mutant *als3 *is not affected by La [52]. Based on information from *Arabidopsis*, it appears likely that the aspen gene identified is functioning in internal Al tolerance. The predicted aspen ALS3 also shared significant sequence identity with STAR2 of rice, which has been suggested to be involved in masking Al binding sites at the cell wall. However, since *STAR2 *is expressed in the root only [21], it seems unlikely that the aspen gene is a homolog of *STAR2*. The second putative aspen Al tolerance gene belongs to the *MATE *gene family. Members of this gene family mediate the release of citrate into the rhizosphere and have been demonstrated to represent major genes of Al tolerance in several plant species [8]. The aspen *MATE *was primarily expressed in the root and was inducible by Al, a pattern comparable to that of the *Arabidopsis MATE *and *MATE *of other plant species [16-20]. Previously, we have shown that aspen releases citrate and oxalate from roots in response to Al exposure. Therefore, it is possible that the aspen *MATE *is involved in the release of citrate and that this mechanism is regulated at least in part at the transcriptional level. An additional aspen gene that may play a role in Al tolerance encodes a pectin methylesterase. This gene was down-regulated early in the response to Al. Pectin methylesterases demethylate pectin and thereby generate free pectin carboxylic groups. The degree of pectin demethylation largely determines the negative charge the pectin matrix carries and thus the amount of Al that can bind to the cell wall. In maize and rice, the degree of pectin methylation has been linked to genotypic differences in Al tolerance. It was demonstrated that the root tips of Al-sensitive lines had a lower degree of pectin methylation and that larger amounts of Al were bound to the cell wall when compared with Al-tolerant lines [11,12]. Consistent with this, higher levels of pectin methylesterase expression were observed in an Al-sensitive maize line than in an Al-tolerant line [26]. Down-regulation of the aspen pectin methylesterase gene thus may serve to reduce Al binding sites at the pectin matrix, and consequently to limit accumulation of Al in the apoplasm.

## Conclusion

This study shows that aspen roots respond to Al exposure with a rapid inhibition of root growth and a large change in gene expression. This early response to Al was characterized by genes involved in cell wall modification, ion transport, and oxidative stress. The subsequent root growth recovery and the concomitant reduction in responsive genes strongly suggest that aspen roots are capable to activate Al tolerance mechanisms when exposed to Al. Based on information from *Arabidopsis *and other plant species, it appears likely that *ALS3, MATE*, and possibly a pectin methylesterase gene are important components of the Al tolerance mechanisms in aspen. These genes and genes with unknown function provide candidates for further studies to elucidate the molecular basis of Al tolerance in aspen.

## Methods

### Plant material

Experiments were performed with *in vitro *propagated plants of the aspen (*Populus tremula *L.) clone Birmensdorf [33]. The plants were maintained in Magenta vessels (GA-7) on 80 ml of 1/2 MS medium (Murashige and Skoog), supplemented with 1% sucrose and solidified with 0.8% agar. The plants were cultivated in a greenhouse and multiplied every 6-8 weeks.

### Solution culture experiments

Aspen roots were treated with Al and La in solution culture prepared in modified Magenta vessels (GA-7). Teflon-racks, placed on the floor of the vessels, supported a 1190 μm nylon mesh (Sefar) as substrate for the plants and two glass slides with a 5 μm grid (N°5, Boraglas) used as a reference to measure root growth. The modified Magenta vessels were sterilized by autoclaving, and filled with 120 ml of autoclaved 1/6 MS solution (pH 4.0) containing 50 nM indol-3-butyric acid (IBA) to induce and synchronize root formation [53]. In each vessel, four cuttings with 3-4 internodes and 1-2 apical leaves were inserted into the mesh so that the roots could form between the wall of the vessel and the glass slide (slotted 1 cm away of the vessel wall). After one day, the nutrient solution was replaced with fresh 1/6 MS medium (pH 4.0) without IBA. The cuttings were incubated without aeration in a climate chamber maintained at 20 ± 2°C with a 16 h light/8 h darkness period (Osram Dulux L 36W/840 color white, Osram). The nutrient solution was replaced twice a week. After 20 d of incubation, after the cuttings had formed 5-15 adventitious roots, the nutrient solution was replaced with treatment solution composed of autoclaved 1/6 MS medium (pH 4.0), supplemented with increasing concentrations of filter-sterilized AlCl_3 _up to 1000 μM (dissolved in 1/6 MS). The pH of the Al treatment solutions was adjusted prior to Al addition with filter-sterilized base (1 M KOH) at amounts empirically determined to ensure that the final pH was 4.0. Treatment experiments with LaCl_3 _were identical to those carried out with AlCl_3_.

The root growth was monitored photographically prior to the treatment (2 d) and during the entire treatment at 12 h intervals and during the first day of the treatment at 6 h intervals. We used a Canon EOS 400 D digital camera fitted with a Canon macro lens EF 100 mm focused on the 5 μm grid of the glass slides. Files were transferred to an Apple MacBookPro, and the pictures were cropped and normalized using the grid on the glass slide with IMAGEJ 1.36b for Macintosh http://rsb.info.nih.gov/ij/. The normalized pictures were used to measure the increase in root length during the particular time intervals. The root growth rate was estimated by dividing each increment by the time elapsed.

Following these treatments, the roots were separated from the shoots and were rinsed in 1/6 MS pH 4.0. The first centimeter of each root was collected, and all the roots processed per plant were transferred to a sterile 1.5 ml tube. The pooled leaves and the stem were collected separately. All the tissues were frozen in liquid nitrogen and stored at -80°C until RNA extraction.

### Quantification of callose

Callose was quantified in the first centimeter of the roots essentially as described by Köhle *et al*. [54]. Ethanol-fixed root tips (four per plant) were blotted dry and homogenized in 0.5 ml of 1 M NaOH with two steel beads in a Retsch MM 200 mixing mill for 3 min. The homogenate was supplemented with 0.5 ml of 1 M NaOH and incubated at 80°C for 30 min. Following centrifugation, 200 μl of the supernatant were mixed with 400 μl of 0.1% (w/v) aniline blue and 1 M glycine NaOH buffer (pH 9.5), and incubated at 50°C for 20 min. Callose was quantified fluorometrically at 400 nm excitation and at 512-521 nm emission wavelength (FluroLog FL3-22, Jobin Yvon), using curdlan as a reference.

### Treatment of plants used for gene expression profiling

Gene expression profiles were determined in a single set of clonal plants grown simultaneously to produce RNA. The plants were treated with no or 500 μM AlCl_3 _for 6 h, 54 h, and 246 h. For each treatment period, three solution cultures were established to allow three independent plants per treatment to be analyzed. Following treatment, the roots were processed as described above and stored at -80°C until RNA extraction. To control the Al concentrations in the nutrient solutions, total Al was quantified by inductively coupled plasma optical emission (ICP-OES; Optima 7300 DV, Perkin Elmer, Wellesley, MA). Prior to analysis, the samples were acidified with nitric acid at 1.5% (v/v).

### RNA isolation

Total RNA from the root tips, stems and leaves were isolated with the Agilent Total RNA Isolation Mini-Kit (Agilent Technologies) according to the manufacturer's instructions. Steps 10 and 11 of the protocol (Agilent Technologies, 2005) were modified as follows: step 10 was carried out twice with 400 μl of wash solution, and step 11 with 400 μl and centrifugation for 1 min, followed by an additional centrifugation for 1 min to ensure that the membrane of the column was completely dry. The concentration of total RNA was determined with a Nanodrop ND-1000 spectrophotometer and the integrity of the RNA was determined with the Eukaryote Total RNA Nano Assay (Agilent Technologies) using the 2100 Agilent Bioanalyzer. Only RNAs with a 260 nm/280 nm ratio between 1.8 and 2.1 and a RNA integrity number between 7 and 10 were processed further.

### Microarray analyses

Microarray analyses were carried out with the Affymetrix GeneChip poplar genome array, which was designed based on sequence information from different poplar species. Synthesis of cDNA, cRNA labeling, and hybridization to the GeneChip poplar genome array were essentially performed as described in the Affymetrix GeneChip Expression Analysis Technical Manual (2005). The cDNA synthesis was performed with 2 μg of total RNA, and the quality of the labeled cRNA was determined using Bioanalyzer 2100.

After hybridization and scanning, probe cell intensities were calculated with the Affymetrix Microarray Analysis Suite (MAS version 5.0) [55]. The Robust Multichip Average (RMA) summary algorithm [56] as implemented in GeneSpring GX 7.3 (Agilent Technologies Inc.) was used to generate and normalize raw gene expression data from probe intensities. Genespring was also used to filter out normalized expression values when not showing present calls in all replicate measurements of at least one condition. To identify genes whose expression differed upon Al treatment at each time point, a Student's t-test was performed in R http://www.r-project.org. To reduce the number of true discoveries that include false positives in multiple and simultaneous statistical tests, a positive false discovery rate (FDR), called *q*, was estimated for each gene. *Q*-values were calculated with the QVALUE software (implemented in R) with the *p*-values (*p *< 0.05, obtained from the t-test) as input and the bootstrap robust settings [34]. Genes were considered to be differentially expressed when (1) the transcript abundance in Al-treated plants was significantly different from that of control plants as determined by the Student's t-test, (2) the calculated *q *was ≤ 0.15, and (3) the change in expression between treated and control plants was at least two-fold. Probe-sets matching the same gene model of the black cottonwood genome sequence http://www.phytozome.net/poplar were declared as redundant. Microarray data are available in the Gene Omnibus Database as accessions GSE19297.

To annotate the expressed transcripts, we performed BLASTX searches against the *Arabidopsis *protein database (TAIR; http://www.arabidopsis.org). The best match was reported, and the resulting list of *Arabidopsis *identifiers was applied to the Functional Catalogue developed by MIPS http://mips.gsf.de/proj/funcatDB to identify biological processes. The MIPS singular enrichment tool was used to identify categories whose members were statistically over-represented compared to the genes present on the microarray. The frequency of genes of a given category on the microarray was calculated as the ratio of the number of genes of this category divided by the total number of genes on the microarray, and the frequency of regulated genes of a given category was calculated as the ratio of the number of regulated genes of this category divided by the total number of regulated genes. Because differentially expressed genes were annotated based on information from *Arabidopsis*, we only considered genes of the microarray representing putative homologs of *Arabidopsis *genes (15,216 genes). Our significance test to search for enriched categories was based on the hypergeometric distribution and is identical to the corresponding one-tailed version of Fisher's exact test, calculating the probability of observing data as extreme or more extreme if the null hypothesis is true. Two strategies to correct for multiple testing were used. The first was Bonferroni's correction, which is a conservative way to control the family wise error rate, and the second a FDR. Functional categories with a *p*-value < 0.05 and a FDR < 0.05 were regarded to be enriched.

### Real-time reverse transcription PCR

Real-time reverse transcription PCR (qRT-PCR) was performed with primers matching aspen sequences that were obtained as follows. Sequence information on the Affymetrix probes was used to design primers, which amplified as long fragments as possible. These primers were applied in RT-PCR and the resulting RT-PCR products were directly sequenced. The aspen sequences obtained were then used to design new primers, which amplified short fragments suitable for qRT-PCR (90-114 bp). When possible, one of the two primers was designed across an exon-exon junction using information from the black cottonwood genome sequence. Primers were designed with the Primer3 software [57]. Sequences of the primers are given in additional file 3.

To validate the results of the microarray analysis, we quantified the expression of eight genes relative to *ACT9*. RT was performed with 200 ng of total RNA using the QuantiTect Reverse Transcription Kit (Qiagen) under conditions recommended by the manufacturer. PCR was performed with the power SYBR green PCR master mix (Applied Biosystems) in a reaction volume of 25 μl containing 5 μl of diluted cDNA. Cycling was carried out on an ABI 7500 Fast real-time cycler (Applied Biosystems) with the following cycling profile: 10 min activation of AmpliTaq Gold Polymerase at 95°C, 45 cycles of 15 sec denaturation at 95°C, 30 sec annealing at 60°C, 30 sec extension at 72°C, followed by a dissociation step of 15 sec at 95°C, 15 sec at 60°C, and 15 sec at 95°C to detect primer dimers and non-specific amplification products. For each primer pair, we determined the PCR efficiency and the dynamic range of PCR by plotting the threshold cycle (C_t_) values generated over a range of dilutions against the log input of cDNA amount. To obtain accurate results, only primer pairs yielding PCR efficiencies of 90-110% (slope of regression between -3.2 and -3.5) were considered [58]. To quantify the transcripts, each cDNA of three biological replicates was diluted in duplicate and used in duplicate PCR. The relative abundance of each transcript was estimated using the ΔΔC_t_ method [59].

The expression of selected genes (*ALS3, MATE*, and *ACT9*) was further quantified by absolute qRT-PCR. Copy numbers of the transcripts were calculated from standard curves that were obtained as follows. Single-stranded sense olignonucleotides specifying amplicons of the selected genes were synthesized (Operon; for sequences see additional file 4). Information from the manufacturer was used to calculate the copy number of the oligonucleotides present in 1 μl of 10 mM Tris-HCl pH 8.5 (Qiagen). Serial dilutions of the stocks were carried out in duplicate, and dilutions in the range from 10^2^-10^8 ^copies were used in duplicate PCR to generate standard curves. The standard curve was obtained by plotting the logs of the calculated copy number against C_t_. The copy numbers of unknown samples were calculated from the regression line. Each cDNA was diluted and run in duplicate, and the transcript copy number was expressed per nanogram of total RNA.

## Authors' contributions

NG carried out the molecular and plant studies, participated in the microarray data analysis, and drafted the manuscript. MGK carried out the screening of the microarrays, SZ performed the statistical analysis of the microarray data, and MM performed the enrichment analysis. TL quantified the callose and participated in the plant studies. IB, LB, and JPM participated in the design of the study and improved the quality of the manuscript. CS coordinated the study and wrote the final manuscript. All authors have read and approved the final manuscript.

## Supplementary Material

Additional file 1 **List of Al-responsive genes in root tips of aspen**. Click here for file

Additional file 2 **Lists of MIPS categories whose genes were significantly enriched (*p *< 0.05; FDR < 0.05)**. Click here for file

Additional file 3 **Primers used to perform real-time reverse transcription PCR in aspen**. Click here for file

Additional file 4 **Sequences of single-stranded sense oligonucleotides specifying amplicons of *ACT9*, *ALS3*, and *MATE *of aspen**. Click here for file
