# Supplementary material for: Transcriptome responses to aluminum stress in roots of aspen (Populus tremula)
Source: BMC Plant Biol. 2010 Aug 23;10:185. doi: 10.1186/1471-2229-10-185 (PMC3017830; doi:10.1186/1471-2229-10-185)
Supplement: Additional file 3 — Primers used to perform real-time reverse transcription PCR in aspen. [file 1471-2229-10-185-S3.DOC]

**Additional file 3** Primers used to perform real-time reverse transcription PCR in aspen. Primers were designed on aspen sequences.

| **Annotation of best hit in *A. thaliana* genome** | **Forward primer (5'-3')** | **Reverse primer (5'-3')** |
| --- | --- | --- |
| Actin 9 (ACT9) | GGT CGT ACA ACT GGT ATC GTG | ACG ACC AGC AAG ATC CAA AC |
| Aluminum sensitive 3 (ALS3) | GCT TAC ATT TTC ATG GTC TCT GT | CCA GCA AGG ATA GAA GCA CCA |
| Basic pathogenesis-related protein 1 (PRB1) | CAT GTG TTG GTG GAG AAT GC | CAT TGC TGC ACC TTG CTT TA |
| F-box family protein (FBL3) | GAA GGG CTT GAC TCC AAG TG | AGA GGC TGT GGA AGA AAC GA |
| MATE (FRD3) | GCT ATG GCT GCA TTC CAA AT | AGC AAA TGC ACC AGC AAT AA |
| Late embryogenesis abundant protein-related (LEA) | AAA ACA AGA AGC AGA AGG GAT G | TAT CCA TTG CAG TTT GGT CTT C |
| Magnesium transporter CorA-like family protein | CCC GTT ACT TTC TTA CCC ATC TAC | AAT TCA ACA ACA AAA CCT CTT GC |
| Peroxidase, putative | GAT TGC TTT GTT AAT GGG TGT G | TGT CAA TAA CTT CGA AAC CTC TCA |
| Sulfate transmembrane transporter (SULTR3;5) | AGC ATG TTT TGC TAG ATT TGA CG | TCA TCT TAA TGT GTT TGA CTT CCA |
