# Supplementary material for: Transcriptome responses to aluminum stress in roots of aspen (Populus tremula)
Source: BMC Plant Biol. 2010 Aug 23;10:185. doi: 10.1186/1471-2229-10-185 (PMC3017830; doi:10.1186/1471-2229-10-185)
Supplement: Additional file 4 — Sequences of single-stranded sense oligonucleotides specifying amplicons of ACT9, ALS3, and MATE of aspen. [file 1471-2229-10-185-S4.DOC]

**Additional file 4** Sequences of single-stranded sense oligonucleotides specifying amplicons of *ACT9*, *ALS3*, and *MATE* of aspen. The oligonucleotides were used to generate standard curves for absolute real-time reverse transcription PCR.

| **Gene** | **Single-stranded sense oligonucleotide sequence (5'−3')** |
| --- | --- |
| *ACT9* | GGTCGTACAACTGGTATCGTGTTGGATTCTGGTGATGGTGTGACTCACACTGTGCCAATCTATGAAGGGTATGCCCTTCCACACGCCATCCTTCGTTTGGATCTTGCTGGTCGT |
| *ALS3* | TGAAATGGCAAGGGAGCAGTATCAATATCTGAAAAATCTACCATGTGATGTGATAGTTTCTGATTGTACAATGCGTAATGCGCAAATGAAATG |
| *MATE* | GCTATGGCTGCATTCCAAATTTGCTTACAAGTTTGGTTGACATCCTCCCTTCTTGCTGATGGCTTGGCAGTTGCCGGACAGGCAATTATTGCTGGTGCATTTGCT |
